# Screening of *BRCA1/2* genes mutations and copy number variations in patients with high risk for hereditary breast and ovarian cancer syndrome (HBOC)

**DOI:** 10.1186/s12885-020-07250-0

**Published:** 2020-08-10

**Authors:** Fatima Zahra El Ansari, Farah Jouali, Nabila Marchoudi, Mohcine Mechita Bennani, Naima Nourouti Ghailani, Amina Barakat, Jamal Fekkak

**Affiliations:** 1 Biomedical Genomics and Oncogenetics Research Laboratory, Faculty of Sciences and Techniques of Tangier, University Abdelmalek Essaâdi, 90000 Tangier, Morocco; 2 Molecular Biology Department, ANOUAL Laboratory, Casablanca, Morocco

**Keywords:** Hereditary breast and ovarian cancer, *BRCA1*, *BRCA 2*

## Abstract

**Background:**

Hereditary breast and ovarian cancer (HBOC) is an autosomal dominant inherited cancer susceptibility disorder. Both *BRCA1* and *BRCA2* genes are considered as high penetrance genes of this syndrome. The identification of *BRCA1/2* genetic alterations before cancer development, grant patients the chance to benefit from various medical cancer prevention approaches. Therefore, the appearance of recent advanced technologies in molecular analysis such as next generation sequencing has simplified full *BRCA1/2* analysis.

Many attempts took place in hope of understanding the molecular germline spectrum of these two genes in Moroccan HBOC patients. However, most of the past projects focused only on young breast cancer cases, lacked ovarian cancer cases in their cohort and only a limited number of these studies were able to analyze the entire exons or copy number variations for both genes.

In attempt of gaining more information regarding the molecular profile of *BRCA1/2* in HBOC, we conducted a study in which we analyze their molecular profile on selected Moroccan patients suspected of having HBOC syndrome.

**Methods:**

In this study we obtained blood samples from 64 selected Moroccan patients, who suffered from Breast and/or ovarian cancer and had a strong family history for cancer. To analyze *BRCA1/2* punctual variants and copy number variations, we used the Ion Personal Genome Machine (PGM) and Oncomine *BRCA1/2* research assay panel. Afterward, we correlated the molecular results with the clinic-pathologic data using IBM SPSS Statistics ver 2.

**Results:**

From the 64 selected cases, Forty-six had breast cancer, fifteen had ovarian cancer and three had both breast and ovarian cancer. The molecular analysis revealed that 18 patients from the 64 harbored a pathogenic variant (28%). Twelve had six different *BRCA1* pathogenic variants and six had six different *BRCA2* pathogenic variants. In this study, we report four pathogenic variants that to the best of our knowledge has never been reported in the Moroccan population before. Regarding copy number variation analysis, No CNV was detected in both genes for all the 64 successfully sequenced and analyzed patients in our cohort.

**Conclusion:**

Work like the present has an important implication on public health and science. It is critical that molecular profiling studies are performed on underserved and understudied population like Morocco.

## Background

Hereditary breast and ovarian cancer (HBOC), is an autosomal dominant inherited cancer susceptibility disorder [1], account for 5–7% of all breast cancer cases [2], and 10–15% of all ovarian cancer cases [3]. The diagnosis of this syndrome has been entrenched in 1970 [4, 5], since then the scientific world has been conducting many studies in hope of defining the clinical characteristics of this syndrome, and understanding its genetic origins.

Until date, the national comprehensive cancer network (NCCN) has established clinical guidelines that help in distinguishing HBOC patients from other sporadic cases [6]. Moreover, genetic studies have demonstrated that HBOC is a highly heterogeneous disease, associated with germline genetic alterations in a number of genes such as *BRCA1, BRCA2, TP53, PTEN, ATM, NBS1, RAD50, BRIP1* and *PALB2* [7]. With BRCA1 and *BRCA2* as main predisposing genes of HBOC. Hence, *BRCA1* and *BRCA2* carriers present a risk of 65–80% and 45–85% for developing breast cancer while, for ovarian cancer they present a risk of 37–62% and 11–23% respectively [8].

The identification of BRCA1/2 genes pathogenic alterations in patients suspected to have HBOC, before cancer development or in the first stages of breast and ovarian cancer, grants them the chance to benefit from various medical cancer prevention approaches, such as risk-reducing surgery (mastectomy and salpingo-oophorectomy), chemo-prevention and enhanced surveillance approaches [9]. Therefore, *BRCA1/2* genetic analysis has become a fundamental concern of doctors and families with a high risk of HBOC syndrome.

Since the beginning of molecular analysis till date, *BRCA1/2* genetic screening has been massively facilitated by the appearance of recent advanced technologies. This advancement has aid enormously in the characterization and the identification of both genes genetic alterations. So far globally, a total of 1826 pathogenic *BRCA* variant has been reported [10]. Other genetic alterations such as large exon, gene deletions/duplication have also been reported in HBOC families with a significant proportion [11].

Although there are similarities among *BRCA1/2* testing criteria worldwide, several reports had shown that different populations have variable *BRCA1* and *BRCA2* mutation spectrum and prevalence [12–14]. Therefore, it is only reasonable to hypothesize that the spectrum and the prevalence of pathogenic variants will differ in Morocco as well. Accordingly, different molecular studies of *BRCA1/2* have been conducted in hope of understanding their molecular germline alterations in Moroccan HBOC patients. Most of these studies have focused only on breast cancer cases and on young patients due to the fact that breast cancer is described as young women disease in the Arab world and other developing nations like some African and Asian countries [15–17]. Moreover, studies that analyzed the molecular profile of *BRCA1/2* in HBOC Moroccan patients rarely had ovarian cancer cases in their cohort. Finally, a limited number of Moroccan studies were able to analyze the entire exons or copy number variations for both genes [18–20].

In attempt of filling some gaps and gaining more information regarding the molecular germline profile of *BRCA1/2* in Moroccan HBOC and around the globe. We initiated a study in which we analyze the punctual variants and copy number variations of both genes on selected HBOC Moroccan patients. To achieve our goal, we used next generation sequencing technology and the commercially available Oncomine *BRCA1/2* research assay panel. This panel analyze both punctual variants and large exons deletions and duplications. Afterward, we correlated the molecular results with the clinico-pathological data of the patients, in hope of understanding more the implication of these molecular alterations in HBOC syndrome.

## Methods

### Study population

A total of 64 female at-risk for HBOC were enrolled in this study between 2016 and 2020. The study material has been collected from individuals referred to ANOUAL laboratory for *BRCA1/2* genetic testing. Even though all cases were recruited from the same city, they had different ethnicity. However, only 23 from the 64 gave their origins the others gave their living location Therefore; this parameter was not included in this study.

All the cases recruited had to fulfill the National Comprehensive Cancer Network (NCCN) selection criteria. HBOC was suspected in cases with breast and/or ovarian cancer, and a personal or a family history of any of the followings:

- Breast cancer diagnosed at or before the age of 45 years,
- Ovarian cancer diagnosed at or before the age of 45 years,
- Multiple primary breast cancers either in one or both breasts,
- triple-negative breast cancer diagnosed before the age of 60 years,
- Two or more relatives with breast or ovarian cancer, with at least one under the age of 50 years;
- Three or more relatives with breast or ovarian cancer at any age.

Prior to the collection of blood samples and clinical data from all participants. Written informed consents were signed from them and their relatives that were recruited in this study. All included Siblings in this study were considered as one case. A pre-analysis genetic counseling was accomplished with patients to collect their family and tumor information. This project was approved by the local Ethics in Research Committee.

### DNA isolation

Peripheral blood samples from the patients were collected in EDTA tubes. Genomic DNA was extracted automatically from blood samples using Maxwell® 16 Blood DNA Purification Kit and stored at − 20 °C. DNA purity and concentration was measured by Nano Drop 2000 Spectrophotometer and Qubit 3.0 (Thermo Fisher Scientific, Waltham, USA).

### Next generation sequencing

*BRCA1* and *BRCA2* genes were screened using the Ion PGM sequencing platform and Oncomine *BRCA1/2* Research Assay panel, this panel contains 265 primer pairs in two pools, covering all coding sequences of both genes, including all splice sites with an average of 64 bp extensions from the intron junctions. The sequencing was carried out in three steps: library preparation, template preparation and finally templates sequencing.

Libraries were generated according to the manufacturer’s instructions guidelines. Briefly, we used 10 ng of DNA isolated from whole blood to generate the sequencing libraries by amplification reaction for each pool. After target amplification, pool 1 and pool 2 amplification reactions were combined into one PCR tube. Followed by partial digestion of primers using FuPa enzyme, and barcode adapter’s ligation. Finally, generated amplicons were purified using AMPure™ XP Reagent. Barcoded purified libraries were quantified and diluted to 100pM before template preparation.

Sequencing templates were produced by clonal amplification of the libraries using the Ion OneTouch 2 System and Ion Torrent PGM Hi-Q VIEW OT2 Kit (Thermo Fisher Scientific, Waltham, MA, USA). The Template-positive Ion SphereTM Particles were isolated with MyOne Streptavidin C1 Beads (Thermo Fisher Scientific, Waltham, MA, USA), and washed with Ion OneTouch Wash Solution, Using the Ion OneTouch ES system (Thermo Fisher Scientific, Waltham, MA, USA). Finally, the barcoded and enriched templates were loaded on the Ion Torrent 316 V2 sequencing chip for deep sequencing on the personal genome machine (PGM) using Ion PGM Hi-Q view Sequencing Solutions kit, all according to the manufacturer’s protocol.

### Data analysis

Generated raw data sequences quality assessment and alignment to the hg19 human reference genome, was carried out by Ion Torrent Suite software (version 5.0.5; Thermo Fisher Scientific). Coverage analysis and Single nucleotide variant calling were performed using Torrent coverage and Variant Caller plug-in (version 5; Thermo Fisher Scientific).

Generated BAM files were used for the annotation of single nucleotide variants, insertions, deletions and copy number variants in the Ion Reporter Server System (Thermo Fisher Scientific) according to the original algorithm pipeline developed by Thermo Fisher Scientific for *BRCA1/2* Research Assay Panel. Only single nucleotide variants with a coverage superior to 250X were considered authentic.

### Genetic variant classification

Genetic variants were classified according to the guidelines of the American College of Medical Genetics and Genomics (ACMG) using a five-tier system: pathogenic, likely pathogenic, variant of unknown significance (VUS), likely benign, or benign [21].

Variants including nonsense, missense, frameshift, large genomic rearrangements, and splice site, were considered as pathogenic when resulting in a prematurely truncated protein and/or reported in BIC or Clinvar as pathogenic. If not their pathogenicity is predicted by in silico analysis via: Sift, Polyphen, Mutation taster and identified through a literature search.

### Statistical analysis

Statistical analysis to assess the association between clinic-pathologic data and *BRCA1/2* punctual variants status was carried out with IBM Statistical Package for Social Science (SPSS) version 20. Chi-square (χ2) test and Fisher’s exact test both were applied to obtain the *P*-value. Correlations was defined as *P* < 0.05.

## Results

### Clinical features of the cohort

From the 64 collected cases, Forty six females had breast cancer fifteen females had ovarian cancer and three had both breast and ovarian cancer. Therefore, in our cohort we have 49 breast tumors and 18 ovarian tumor.

The median age of all the recruited patients in our cohort was 42, ranging from 23 to 55 years old. The majority of our BC tumors had Ductal carcinoma (91%), T2 tumor size (44.8%), SBR grade II (46.9%), and luminal subtype of cancer (59%). All the clinico-pathological data of breast cancer cases are summarized in Table 1. Regarding ovarian cancer cases, the majority had serous carcinoma (66.67%), T3 tumor size (61.11%) and SBR grade III (55.56%). All the clinico-pathological data of ovarian cancer cases are listed in Table 2.

**Table 1:** Correlation of the clinical characteristics with mutation status in Breast cancer cases

clinical characteristics	Total	***BRCA1/2*** Carriers	non Carriers	***P***-value
**Histological type**				0.8
ductal carcinoma	45 (91.8%)	12 (92.3)	33(91.6)	
lobular carcinoma	3 (6.1%)	1(7.6)	2(5.5)	
Others	1 (2.04%)	0	1(2.7)	
**Tumor size**				0.7
T1	14(28.5%)	3(23.0%)	11(30.5%)	
T2	22(44.8%)	5(38.4%)	17(47.2%)	
T3	11(22.4%)	4(30.7%)	7(19.4%)	
T4	2(4.08%)	1(7.6%)	1(2.7%)	
**SBR grade**				0.9
I	6(12.2%)	2(15.3%)	4(11.1%)	
II	23(46.9%)	6(46.1%)	17(47.2%)	
III	20(40.8%)	5(38.4%)	15(41.6%)	
**Molecular type**				0.03
triple negative	17 (34.6%)	8 (61.5%)	9 (25.0)	
luminal	29 (59%)	5 (38.4%)	24(66.6)	
HER2+	3 (6.1%)	0(0%)	3(8.3)

**Table 2:** Correlation of the clinical characteristics with mutation status in ovarian cancer

clinical characteristics	Total	BRCA carriers	BRCA non carriers	***P***-value
**Histological type**				0.73
**Serous**	12(66.67%)	4(71.42%)	7(63.64%)	
**Other**	6(33.33%)	2(28.57%)	4(36.36%)	
**Tumor size**				0.47
**T3**	11(61.11%)	4(71.42%)	7(54.54%)	
**T4**	7(38.89%)	2(28.57%)	5(45.45%)	
**SBR grade**				0.38
**II**	8(44.44%)	4(57.14%)	4(36.36%)	
**III**	10(55.56%)	2(42.86%)	8(63.64%)	

### Pathogenic variants identified in *BRCA1* and *BRCA2*

After performing *BRCA1/2* molecular analysis, we detected 12 pathogenic variants in 18 patients from 64 (28.%). 12 patients harbored *BRCA1* variant, and 6 harbored *BRCA2* variant.

In *BRCA1* gene we detected 6 different pathogenic variants and in *BRCA2* gene we detected 6 different pathogenic variants as well. All the pathogenic variants found in our study are reported in Table 3. All these variants were stated in both BIC and Clinvar.

**Table 3:** Detected BRCA1/2 pathogenic variants

Gene	DNA level (Protein level)	NM	Families	Exon	Variant type	Molecular consequence	Protein consequence	Variant coverage
BRCA1	c.798_799delTT (p.Ser267fs)	3	I,II,III	11	Deletion	frameshift	Premature stop codon at p.Ser267Lysfs*19	834
								1007
								768
	c.3279delC (P.Tyr1094fs)	4	IV,V,VI	11	Deletion	framshift	Premature stop codon at p.Tyr1094Ilefs*15	531
								468
								679
								788
	c.4823C > G (p.Ser1608Ter)	1	VII	16	SNV	nonsense	Premature stop codon atp. Ser1608Ter	1340
	c.1016dupA (p.Val340fs)	1	VIII	10	duplication	frameshift	Premature stop codon atp. Val340Glyfs*6	543
	c.66_67delAG (p.Glu23fs)	2	IX, X	2	Deletion	frameshift	Premature stop codon atp. Glu23fs*17	634
								957
	c.5158C > T (p.Arg1720Trp)	1	XI	18	SNV	missense	Premature stop codon atp. Arg1720Trp	729
BRCA2	c.1302_1305delAAGA (p.Lys437fs)	1	XII	10	Deletion	frameshift	Premature stop codon atp. Lys437fs*22	934
	c.7110delA (p.Lys2370fs)	1	XIII	14	Deletion	frameshift	Premature stop codon atp. Lys2370fs*	1021
	c.3847_3848delGT (p.Val1283fs)	1	XIV	11	Deletion	frameshift	Premature stop codon atP.Val1283fs*2	670
	c.5576-5579delTTAA (p.I1859fs)	1	XV	11	Deletion	frameshift	Premature stop codon atp. I1859fs*3	534
	c.7235_7236insG (p.Lys2413fs)	1	XVI	14	insertion	frameshift	Premature stop codon atp. Lys2413fs*	760
	c.3860delA (p.Asn1287fs)	1	XVII		Deletion	frameshift	Premature stop codon atp. Asn1287fs*6	1167

From the 18 patients with pathogenic variants in *BRAC1/2* genes in our study; twelve had breast cancer, five had ovarian cancer and one had both breast and ovarian cancer. The breast data of the last mentioned patient were included in Table 1 and her ovarian data were included in Table 2.

### Variants of uncertain significance in *BRCA1* and *BRCA2*

From all the 64 successfully sequenced patients we found one uncertain significance variant in one ovarian cancer patient and her sister that suffered from breast cancer. This variant was not found in their healthy third sister. The variant c.91 T > G (p.Trp31Gly) is a substitution of the nucleotide thymine with guanine in exon 3 of *BRCA2* gene, causing the substitution of Tryptophan to glycine in the binding region of *BRCA2* gene. This variant was included in the statistical analysis between BRCA carriers and no carriers in Table 2. No likely pathogenic variant was detected in our population.

### Copy number variations

No CNV was detected in both genes for all the 64 successfully sequenced and analyzed patients in our cohort.

## Discussion

To date, there have been several attempts to describe the spectrum of *BRCA1/2* germline pathogenic variants in Moroccan patients with HBOC syndrome. However, past works have failed to depict a comprehensive picture; they either focused only on young patients with breast cancer, lacked ovarian cancer cases in their cohort, or only sequenced selected regions of both genes. Moreover, no Moroccan study has analyzed copy number variations for both genes in patients suspected for HBOC before. Thus, to overcome past limitations, the goal of our study is to describe the punctual variants and copy number variations profile of *BRCA1* and *BRCA2* genes on Moroccan patients with breast and/or ovarian cancer suspected to have HBOC syndrome.

Respecting the National Comprehensive Cancer Network (NCCN) criteria for HBOC syndrome, we were able to recruit 64 patients suspected of HBOC syndrome; Forty-six (71.9%) had breast cancer, fifteen (23.4%) had ovarian cancer and three (4.7%) had both breast and ovarian cancer. The predominance of breast cancer cases in HBOC cohort is reported in all HBOC studies worldwide [22, 23]. Epidemiological studies in Morocco have shown that the frequency of breast cancer is higher than ovarian cancer, which increases the probability for the predominance of breast cancer patients in our cohort [19, 24, 25].

In agreement with other studies, the median age of all the 64 analyzed patients in our cohort is 42 years old. For breast cancer cases, alone the median age is 42 years old, and for ovarian cancer cases alone the median age is 43 years old. To the best of our knowledge, no Moroccan study has worked on HBOC cohort with ovarian cancer cases before. Therefore, we are only able to compare our breast cancer cases median age with the one reported by Tazzite et al. [26] and Laraqui et al. [19]. Our findings correlate with both studies that reported a median age inferior to 45 years old for familial breast cancer cases. On the other hand, our entire HBOC cohort median age line up with that reported by Ciernikova et al. [22] and Alemar et al. [23]. However, our cohort median age is still younger than the one reported by Tingyan Shi et al. [27]. Many studies have indicated that the median age of onset in north African countries including Morocco is more than 10 years younger than the age of onset in European/North American countries [15, 26].

Clinico-pathological data of all patients included in this study, were collected through a review of the patients medical records. The majority of our BC cases had Ductal carcinoma (91%), T2 tumor size (44.8%), SBR grade II (46.9%), and luminal subtype of cancer (59%). These results correlate with other local and international studies [28–30]. Besides, according to Tazzite et al. [26] and Musolino et al. [31], these characteristics are also the most encountered in sporadic breast cancer cases. Apropos ovarian cancer cases, the majority had Serous carcinoma (66.67%), T3 tumor size (61.11%), and III SBR grade (55.56%). We didn’t find any Moroccan study concerning ovarian cancer clinico-pathological data. However, Our findings line up with other studies on other populations [32, 33].

In this study, the molecular analysis was performed using the Oncomine *BRCA1/2* Research Assay Panel that could detect both punctual variants and copy number variations. It is considered as an alternative assay to investigate both types of *BRCA1/2* genetic alterations in one workflow. This panel efficiency has been investigated by Hirotsu et al. (2017) [34], and according to his study results, the Oncomine *BRCA1/2* research assay panel is a highly accurate tool for analyzing variants with wide-ranging allelic fractions and for detecting copy number alterations.

To the best of our knowledge, this is the first Moroccan study to analyze *BRCA1/2* copy number variations in Morocco. Globally, only a limited number of studies analyzed *BRCA1/2* CNV profile in HBOC patients. From a population to another the results are still contradicted [35, 36], which emphasize the importance of more studies to build solid conclusions regarding the implication of *BRCA1/2* CNV in HBOC.

After molecular analyses of *BRCA1/2* genes*,* eighteen patients (28.1%) in our cohort from the 64 harbored *BRCA1/2* pathogenic alteration. In *BRCA1* gene we found 6 different pathogenic variants in 12 patients (66%). while for *BRCA2* gene we found 6 different pathogenic variants in 6 patients (33%) and one variant of uncertain significance. No copy number variation was detected in both genes for all the successfully sequenced 64 patients. All the pathogenic variants detected in our study and their molecular effect are summarized in Table 3 and are mentioned in both data bases BIC and Clinvar. The prevalence of *BRCA1/2* pathogenic variants that we found in this study line up with that reported by Laraqui et al. [37], Jouali et al. [38] and Alemar et al. [23]. However, this prevalence is still higher than that reported by Tazzite et al. [18]. This variability in pathogenic punctual variants percentage could be explained by the fallowed selection criteria and the technology used in these studies. In our cohort, we found a dominance of *BRCA1* gene pathogenic variants compared to *BRCA2* pathogenic variants these results correlate with other studies [23, 37].

Regarding *BRCA1* gene we found 6 different pathogenic variants. The first is c.798_799delTT (p.Ser267fs), it was detected in three unrelated patients. All three had breast cancer from which two had triple negative subtype and one had luminal subtype. This variant has been reported in different Moroccan studies [38, 39] and also was reported in some Tunisian and Algerian studies, to be the first non-Jewish founder *BRCA*1 pathogenic variant in north Africa [37].

Another four patients harbored the variant c.3279delC (P.Tyr1094fs), two were discovered to be second degree relatives from the mother side after investigation. Both cousins had luminal breast cancer. As for the third patient, she suffered from aggressive triple negative breast cancer and ovarian cancer. Finally, the last patient suffered from triple negative breast cancer. This variant was first reported in 2005 by van der Hout et al. [40] in Netherlands, then it was reported for the first time in morocco at 2012 by Tazzite et al. [18], afterward it was reported by El khachibi et al. [41] and in 2015 it was reported by Strom et al. [42] in the USA. The high prevalence of this variant in our cohort and the fact that it has been reported several times in morocco state the probability of it being a founder mutation of our population or maybe for a specific ethnicity in morocco. However, none of the four patients provided their origins and we were unable to contact them.

The third variant C.4823C > G (p.Ser1608Ter) was detected in one patient and her sister. Both came for *BRCA1/2* testing after one was diagnosed with triple negative breast cancer and the other had ovarian cancer. As far as we know, this variant has been reported in USA and UK by Strom CM et al. [42] and Robertson L et al. [43], and this is the first study to report this pathogenic variant in Morocco.

The fourth *BRCA1* pathogenic variant c.1016dupA (p.Val340fs) was detected in a patient suffering from luminal breast cancer. This pathogenic variant is reported as the 12th most common frameshift variant occurring in *BRCA1* gene. It was detected in different populations [42, 44–46] and once in Morocco by Laraqui et al. [8].

The fifth *BRCA1* pathogenic variant is c.66_67delAG (p.Glu23fs), it was detected in two patients one with triple negative breast cancer and the second with ovarian cancer. It is also known as 187delAG, this variant is one of three main pathogenic founder variants in the Ashkenazi Jewish population [47]. In Morocco, this variant has been reported by Zoure et al. [48] and Jouali et al. [38] and globally, it has been found in different populations [49–51].

The last *BRCA1* pathogenic variant we found in this study is c.5158C > T (p.Arg1720Trp), it was detected in a patient with ovarian cancer. This variant has been reported in Morocco by Laraqui et al. [8], in Finland by Kuusisto et al. [52]^.^and in Italy by Antonucci et al. [53].

Apropos *BRCA2* gene, we identified 6 different pathogenic variants in 6 unrelated patients and one variant of uncertain significance in one patient and her sister. The first patient had ovarian cancer and harbored c.1302_1305delAAGA (p.Lys437fs) pathogenic variant. This variant was identified in morocco by Laarabi et al. [39] and it was reported in several populations around the world [54, 55].

The second patient had triple negative breast cancer and harbored c.7110delA (p.Lys2370fs) pathogenic variant. This variant was reported for the first time by Tazzite et al. [18] and our study is the second in the world to report this variant.

The third patient also had triple negative breast cancer and harbored the pathogenic variant C.3847delG (P.Val1283fs). as far as we know, this is the first Moroccan study to report this variant. Nevertheless, it was reported in different populations worldwide [56–58].

The fourth pathogenic variant C.5576-5579delTTAA (P.I1859fs) was detected in a patient with luminal breast cancer. According to our literature search, this is the first study to report this variant in Morocco, but it has been reported in other studies [59, 60].

Our fifth *BRCA2* pathogenic variant is c.7235_7236insG (p.Lys2413fs) it was detected in a patient with ovarian cancer. This variant has been reported for the first time by Tazzite et al. [18] and our study is the second to report it.

Our last *BRCA2* pathogenic variant is c.3860delA (p.Asn1287fs). It was detected in a patient suffering from ovarian cancer. To the best of our knowledge, this is the first Moroccan study to report this variant however, it was reported in different populations worldwide [61, 62].

Finally, in our study we reported one variant of uncertain significance c.91 T > G (p.Trp31Gly) in *BRCA2* gene. This variant was found in our patient and in one of her two sisters as well. Both sisters carrying the variant suffered from cancer while their third sister who didn’t harbor the variant was healthy. This variant was reported by Laura Caleca et al. [63]. In her study, Caleca proved that this variant affects the binding of BRCA2 protein with the PALB2 protein.

Comparing our *BRCA1/2* identified pathogenic variants with all the pathogenic variants found by other Moroccan studies (Table 4). We detected In *BRCA2* gene, two variants that have been reported only in this study and by Tazzite et al. [18]. The variant c.7235_7236insG (p.Lys2413Terfs) and the variant c.7110del (p.Lys2370fs). The fact that these variants were never reported in other studies worldwide, rise the probability of them being specific pathogenic variants for the Moroccan population. Furthermore, we found four pathogenic variants that have never been reported in other Moroccan studies. The first C.4760C > G (p.S1587Ter) was found in a patient that confirmed her Moroccan Arab origins. The second C.3847delG (P.Val1283fs) was found in a patient that confirmed her Moroccan Amazigh origins. While the third C.5576-5579delTTAA (P.I1859fs9) and the fourth c.3860delA (p.Asn1287fs) were found in patients with European ancestors. However, all four mutations were reported in European studies. We believe that the history of the migration flow between the two populations can explain the origins of these pathogenic variants.

**Table 4:** BRCA1/2 reported pathogenic variants in the Moroccan population

Study	Number of cases	Reported variants			Methodology
		Genetic variant	Consequence	Gene	
Tazzite et al. [18] (Morocco)	40	c.5558dup	p.Tyr1853Ter	BRCA1	Full BRCA1/BRCA2 screening using Sanger
		c.798799delTT	p.Ser267LysfsX1	BRCA1	
		c.2805delA	p.S896Vfs104	BRCA1	
		c.3279delC	p.Ile1859LysfsX3	BRCA1	
		c.5062 5064delGTT	p.Val1688del	BRCA1	
		c.3381delT	p.Phe1127LeufsX	BRCA2	
		c.7110delA	p.Lys2370fs	BRCA2	
		c.7235insG	p.Thr2412fsX2	BRCA2	
		c.7110delA	Lys2370fs	BRCA2	
Laraqui et al. [9] (Morocco)	121	c.798799delTT	p.Ser267LysfsX1	BRCA1	Full BRCA1 gene sequencing using Sanger
		c.1016dupA	p.Lys1698X	BRCA1	
		c.4942A > T	p.Lys1648X	BRCA1	
		c.5095C > T	p.Arg1699Trp	BRCA1	
Laarabi et al. [39] (Morocco)	74	c.68_69delAG	p.Glu23fsX17	BRCA1 BRCA2	51 underwent sanger sequencing for exon 10 of BRCA2 gene. Full BRCA1/2 sequencing for 23
		c.5073dupA	p.Trp1692Metfs		
		c.1310_1313delAAGA	p.Lys437IlefsX22		
Jouali et al. [38] (Morocco)	15	c.2126insA	p.Phe709TyrfsX3	BRCA1	Full BRCA1/BRCA2 screening using next generation sequencing
		c.7234_7235insG	p.Thr2412Serfs	BRCA2	
		c.3453delT		BRCA1	
		c.1310_1313delAAGA	p.Lys437IlefsX22	BRCA2	
Our study	64	c.798_799delTT	p.Ser267LysfsX19	BRCA1	Full BRCA1/BRCA2 punctual variants and copy number variations screening using next generation sequencing
		c.3279delC	p.Tyr1094IlefsX15	BRCA1	
		c.4823C > G	p.Ser1608Ter	BRCA1	
		c.1016dupA	p.Val340GlyfsX6	BRCA1	
		c.66_67delAG	p.Glu23fsX17	BRCA1	
		c.5158C > T	p.Arg1720TrpX	BRCA1	
		c.1302_1305delAAGA	p.Lys437fsX22	BRCA2	
		c.7110delA	p.Lys2370fsX	BRCA2	
		c.3847_3848delGT	P.Val1283fsX2	BRCA2	
		c.5576-5579delTTAA	p.I1859fsX3	BRCA2	
		c.7235_7236insG	p.Lys2413fsX	BRCA2	
		c.3860delA	p.Asn1287fsX6	BRCA2	

In our study, we didn’t report any copy number variation in both genes. All our patients were successfully sequenced and according to the Ion reporter pipeline, the CNV analysis was successful for all the patients in our cohort. Copy number variation in both genes was reported by Wen-Ming Cao et al. [35] with a percentage of 16.1% (5/31). While, other studies state that BRCA1/2 CNVs for HBOC patients is rarely found [64, 65]. More global studies are required to define the implication of *BRCA1/2* CNVs in HBOC.

After molecular analysis, we compared the histopathological characteristics between *BRCA1/2* pathogenic mutations carriers and no carriers for breast and ovarian cancer cases separately. For both cancers, no significant difference was found concerning Histological type, Tumor size, and SBR grade. While, for the molecular subtype of breast cancer we found a correlation between *BRCA1/2* carriers and triple negative breast cancer with a *P*-Value < 0.05. Our results correlate with those of Alemar et al. [23], Cao et al. [66]. Moreover, according to different epidemic molecular studies, *BRCA1* mutation carriers have more chances of developing triple negative breast cancer subtype [67–69].

In our cohort 46 patients (34 BC, 10 OC and 2 BC + OV) didn’t harbor any *BRCA*1/2 pathogenic variant or copy number variation, despite having a strong family history for cancer. These results drive us to suspect that these patients may harbor genetic alterations in other genes implicated in HBOC syndrome such as *TP53, PTEN, ATM, NBS1, RAD50, BRIP1 and PALB2,* which emphasize the need for analyzing all the genes implicated in HBOC in one workflow instead of only *BRCA1/2*.

## Conclusion

Works like the present have an important implications in both public health and science. First, proper risk assessment including genetic testing of high risk individuals can lead to increased awareness of cancer risk and effective use of interventions to reduce *BRCA-*related cancer incidence and mortality. Second, understanding the spectrum of ethnic-specific mutation landscape can lead to genetic tests tailored to ethnic groups, which can increase sensitivity and specificity of analytic techniques as well as lowering the cost. Lastly, genetic testing can indicate presence or absence of *BRCA* mutation as well as variants of uncertain clinical significance (VUS). As catalogue of genetic variants are collected, further refinement is possible to decipher the mechanistic meaning of the VUSs. To achieve a comprehensive collection of all variants worldwide, it is critical that molecular profiling studies are performed on underserved and understudied population such as Morocco. Thus, despite the lack of novelty in our study, we believe that the clinical, economic, and scientific implication of our results is broad and profound.

## Data Availability

The datasets analyzed during the current study and a list of material requirement will be available from the corresponding author on reasonable request.

## Abbreviations

HBOC: Hereditary breast and ovarian cancer
BC: Breast cancer
OV: Ovarian cancer
NCCN: National comprehensive cancer network
CNV: Copy number variation
VUS: Variant of uncertain significant
